# Anti-Yo-Associated Paraneoplastic Cerebellar Degeneration: Case Series and Review of Literature

**DOI:** 10.7759/cureus.20203

**Published:** 2021-12-06

**Authors:** Mathew Chatham, Polly Niravath

**Affiliations:** 1 Medicine, Brophy College Preparatory, Phoenix, USA; 2 Hematology/Oncology, Houston Methodist Hospital, Houston, USA

**Keywords:** paraneoplastic neurologic syndrome, anti-yo antibody, paraneoplastic cerebellar degeneration, breast cancer, gynecologic cancer, paraneoplastic syndrome

## Abstract

Anti-Yo-associated paraneoplastic cerebellar degeneration (PCD) syndrome is a very rare condition that is most commonly associated with breast and gynecologic cancers. Those cases associated with breast cancer tend to be human epidermal growth factor receptor 2 (HER2)-positive, though the reason for this correlation is unknown. Most commonly, the neurologic symptoms of the PCD syndrome predate the patient’s cancer diagnosis. Thus, prompt diagnosis of PCD is essential to allow for early treatment of the neurologic symptoms and the underlying malignancy. However, the prognosis is very poor for the anti-Yo-associated paraneoplastic syndrome, since neurologic damage is usually rapid and irreversible. Further progression may be stopped with appropriate treatment of cancer, but existing neurologic deficits at the time of diagnosis are usually permanent. Steroids, plasma exchange, and rituximab are commonly used treatments, though these have had mixed to poor results.

## Introduction

Paraneoplastic syndromes are rare with <10% of cancer patients diagnosed with a paraneoplastic disorder, most commonly endocrine or neurologic [1]. Neurologic paraneoplastic syndromes (NPS) are particularly clinically relevant because 80% of patients may present with neurologic symptoms prior to being diagnosed with cancer [2]. While an earlier diagnosis of the syndrome is important because it allows for the earlier institution of cancer treatment, the immune-mediated damage of NPS is usually irreversible. Thus, early diagnosis and treatment of cancer may stop the progression of the neurologic disorder, but it typically does not reverse the deficits which may have already developed [3]. Paraneoplastic cerebellar degeneration (PCD) is a rare type of NPS that affects less than 1% of all cancer patients. PCD is most commonly associated with breast, lung, lymphoma, and ovarian cancers. Paraneoplastic cerebellar degeneration is the result of unique antigens present in the tumor, which are also expressed in cerebellar tissue. These antigens stimulate the production of antibodies that attack the Purkinje cells in the cerebellum, causing gait ataxia, dizziness, nausea, dysarthria, nystagmus, and occasionally double vision [4]. 

Of the roughly 30 different antibodies that are associated with PCD, anti-Yo, anti-Tr, and anti-mGluR1 antibodies are the most commonly diagnosed entities. Anti-Yo antibody, also known as Purkinje cell cytoplasmic antibody type 1 (PCA1), accounts for roughly half of the antibody-mediated PCD cases but still proves to be very rare. A study regarding anti-Yo-associated PCD found that only 2.3% of 557 patients with ovarian cancer and 1.6% of 253 patients with breast cancer tested positive for the anti-Yo antibody, and only about 12% of those positive for the antibody had neurologic symptoms of PCD [4]. Anti-Yo-mediated PCD is predominantly associated with ovarian and breast cancers. Therefore, most patients with anti-Yo PCD are women, approximately 60 years old and older, with less than 20 cases found in male patients [5]. Approximately 2% of anti-Yo antibody cases are not associated with known cancer, including some patients in whom an autopsy also did not reveal underlying cancer, despite the presence of PCD syndrome and anti-Yo antibodies in the serum [4,6].

In this review, we will discuss three cases of anti-Yo paraneoplastic neurologic syndrome in patients with breast and suspected ovarian cancers, including their clinical presentations, treatment, and survival.

## Case presentation

Case 1

Case 1 is a 57-year-old healthy woman with a past medical history significant only for hypertension. Her family history is notable for a sister with estrogen receptor-positive (ER+), progesterone receptor-positive (PR+), human epidermal growth factor receptor 2 (HER2) breast cancer at the age of 57 years, and a maternal aunt with breast cancer at the age of 54 years. The patient’s germline panel genetic testing was negative. She began having poor coordination in the right foot in September 2017; she noticed this suddenly, at a moment when she was attending a baseball game. Her coordination worsened rapidly and globally over the next several months to the point that she was unable to walk or write by December 2017. She also developed dysarthria and she lost 25 pounds over three months. She initially had a negative workup, including brain MRI, CT chest/abdomen/pelvis, lumbar puncture, and serologic tests for syphilis, HIV, and vitamin B12. Her neurologist sent a paraneoplastic antibody panel from her serum and found her to be positive for anti-Yo antibodies. She presented to our facility in March 2018. The mammogram showed a left breast mass and left axillary lymphadenopathy. Lymph node biopsy revealed adenocarcinoma, consistent with breast primary, GATA3 positive, estrogen receptor-negative, progesterone receptor-negative, HER2-positive (3+ on immunohistochemistry {IHC}), and Ki-67 of 35%. 

In late December 2017, she was treated with steroids and IVIG for the anti-Yo antibody syndrome. She had minimal improvement in her dysarthria but no change in her ataxia and poor coordination. She then underwent left modified radical mastectomy on April 2, 2018, which showed 4.7 cm grade 2 invasive ductal carcinoma, with eight of 17 lymph nodes positive for breast cancer. Due to her performance status of 4, and a poor chance of neurologic recovery, the tumor board did not recommend chemotherapy or radiation for her. Rather, the board recommended a less conventional approach of only trastuzumab and pertuzumab for 17 cycles, which she completed in March 2019. This treatment was felt to be more tolerable and safe for her than the standard chemotherapy plus antibody approach. She remains in remission from her breast cancer, now 39 months from her diagnosis. For her paraneoplastic cerebellar ataxia, she did receive rituximab from March 2019 through October 2020, with a minimal improvement of her symptoms. She also received two courses in plasma exchange. After the first session of plasma exchange treatments, her PCA-1 titer in the cerebrospinal fluid (CSF) fell from 1:256 to 1:64. However, after a later series of plasma exchange treatments, her CSF PCA-1 titer rose to 1:128 in July 2021. The neurologist noted that these titers did not correlate with her clinical symptoms. She continues to do occupational therapy at home, but she is still wheelchair-bound and needs assistance with all activities of daily living, including dressing, bathing, and feeding herself. She remains dysarthric.

Case 2

Case 2 is a 61-year-old woman with pre-diabetes and hyperlipidemia who presented with vertigo, nausea, vomiting, and ataxia beginning in March 2018. She had a negative MRI brain, magnetic resonance angiography (MRA), and carotid ultrasound at that time. Then, in June 2018, her symptoms progressed to left upper extremity weakness, diplopia, 20-pound weight loss, and worsening ataxia. She was then found to be positive for anti-Yo antibodies with a serum titer of 1:61440. CT scan of the body showed only 1.9 cm left axillary lymphadenopathy; biopsy of this lymph node was positive for metastatic grade 3 breast carcinoma, GATA3-positive, thyroid transcription factor 1 (TTF1)-negative, SRY-related high mobility group box 10 (SOX10)-negative, estrogen receptor-negative, progesterone receptor-negative, HER2-positive (2+ on immunohistochemistry {IHC}, fluorescence in situ hybridization {FISH} ratio of 5.6), and Ki-67 of 80%. In July 2018, she underwent a left modified radical mastectomy, which showed no cancer or atypia in the breast, and four of 14 lymph nodes with high-grade metastatic breast carcinoma. This serves as one of the rare reports of anti-Yo-associated PCD associated with axillary HER2-positive cancer, with absent or occult breast primary. She received 17 cycles of trastuzumab and pertuzumab from July 2018 through July 2019. Chemotherapy was omitted for case 2 due to poor performance status, similar to the reasoning for case 1. For treatment of her paraneoplastic syndrome, she received plasma exchange from July 2018 through February 2019. She then received rituximab from February 2019 through December 2019. Her serum antibody titer became as low as 1:240 in November 2018. However, it began to rise again, up to 1:15360 in December 2018. Her antibody titers over time did not correlate with her neurologic symptoms. She remains in a wheelchair, but she is able to take a few steps, with assistance. She also has dysarthria and poor coordination. She requires full assistance with her activities of daily living. She remains in remission, 36 months from the time of her diagnosis. 

Case 3

**Figure 1:**
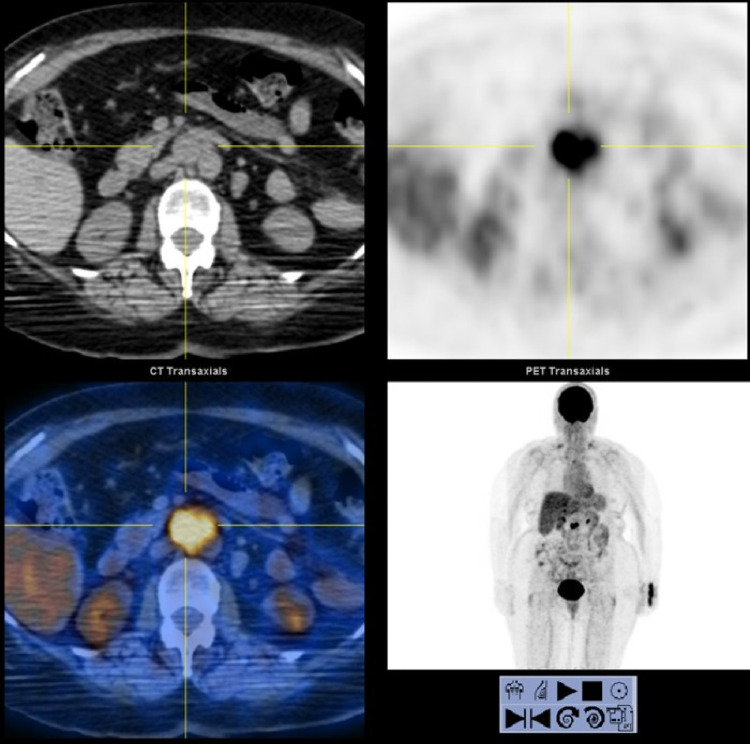
Positron emission tomography (PET) scan from case 3 showing para-aortic lymphadenopathy, with standard uptake value (SUV) 18.5

Case 3 is a 79-year-old woman who presented in August 2016 with four months of progressive ataxia, worsening vision, and fatigue. She was unable to walk when she presented to her neurologist. A PET/CT in August 2016 showed only a 3 cm para-aortic/aortocaval lymphadenopathy (LN), with standard uptake value (SUV) of 18.5 (Figure 1). 

This lymph node was not accessible for percutaneous biopsy. Her mammogram and breast ultrasound were negative. Her CA-125 was elevated at 1048. She did not have a suitable target for tissue biopsy, and she declined chemotherapy. She received radiation therapy to the aortocaval lymph node in January 2017, with subsequent improvement seen on the PET scan. She was presumed to have ovarian cancer, though a tissue diagnosis was never obtained. Her paraneoplastic panel showed anti-Yo antibodies, and she was diagnosed with paraneoplastic cerebellar degeneration syndrome. She was initially started on plasma exchange and steroids, with some clinical improvement. She then later tried a brief course of IVIG, with no significant improvement. She declined systemic therapy, and she progressed rapidly, to the point where she was totally unable to speak or walk by April 2017. She was also having some difficulty with swallowing solids and liquids, beginning to choke more frequently at meals. She declined hospice and was lost to follow-up after April 2017. 

Treatment

With the rarity and varying prognosis of PCD, treatment for anti-Yo antibody-mediated paraneoplastic syndrome remains uncertain. However, because the syndrome is immune-mediated, the treatments are typically immunosuppressive. Steroids have shown limited to no success in case reports, with only minimal and ephemeral response seen in one of 17 patients in one report [6]. Similarly, chemotherapy medications like cyclophosphamide have not shown significant success [4], except for rare case reports [7]. Rituximab has also been used, though less commonly, in some patients, with very low rates of response [8]. 

IVIG is another treatment that may also be used in the treatment of PCD, though it also has shown mixed results, and the mechanism of this treatment is poorly understood. However, the reports of IVIG efficacy tend to be associated with earlier treatment, ideally within one month from the time of symptom onset [9]. Another method that has seen used to treat patients with anti-Yo-mediated PCD is plasma exchange. Since the antibody is primarily found in plasma, this treatment aims to remove the agents through the removal of the affected plasma. Several case reports have shared extremely successful results from plasma exchange [5,10], while others have been mostly unsuccessful [6]. 

Treatment of cancer remains the mainstay of treatment for anti-Yo antibody paraneoplastic cerebellar degeneration, though this may only stop further progression; it is highly unlikely to reverse cerebellar damage which has already occurred.

## Discussion

As illustrated by the three cases above, anti-Yo antibody-associated paraneoplastic cerebellar degeneration syndrome tends to be rapidly progressive and irreversible, often resulting in severe morbidity, to the point of complete dependence on others and inability to walk or perform most activities of daily living. For example, in a report by Peterson et al., only 3.6% of individuals with PCD were able to walk without assistance as a result of the severe ataxia [6]. Thus, earlier diagnosis and prompt treatment of initiation are important, as they may prevent worsening neurologic deficits but cannot reverse the deficits which have already occurred. Furthermore, even with successful treatment of cancer, many patients have persistent anti-Yo antibodies in serum or CSF, though cancer may have been in remission for years. There has never been a clear correlation between antibody titers and the severity of neurologic symptoms. Thus, it is not recommended to routinely check antibody levels during treatment. For example, antibodies may decline during plasma exchange therapy, but symptoms may not improve [6]. 

Interestingly, for those breast cancer cases associated with anti-Yo PCD, a large number of them are associated with HER2-positive breast cancer. While only 15-25% of all breast cancer overexpresses HER2, a series of breast cancer patients with anti-Yo antibodies has shown that up to 96% of these patients have HER2-positive breast cancer. This same over-representation of HER2-positive breast cancer is not seen in other PCD syndromes mediated by other antibodies [11]. It is not known why anti-Yo antibody-associated PCD syndrome is associated with HER2-positive breast cancers, as opposed to other types of breast cancers. In one study, less than a quarter of the HER2-positive breast cancer cases associated with anti-Yo PCD expressed cdr2, which is the onconeural antigen that is recognized by anti-Yo antibodies. There are no reports on HER2 expression in gynecologic cancers associated with PCD, as the HER2 marker is not typically tested in gynecologic cancers.

The prognosis of anti-Yo-mediated PCD is usually unfavorable with long-term survival rates being below 25% [6]. Despite the numerous proposed treatments for the condition, all the known treatments for anti-Yo-mediated PCD have served primarily to lessen the severity of symptoms and limit their progression. However, the antibody-mediated damage to the cerebellum cannot be reversed, leaving patients with permanent, severe neurological impairment [4]. In long-term follow-up, less than 10% of patients are able to ambulate without assistance with the majority of patients left bedridden [11]. Median overall survival for patients with breast cancer has been found to be 100 months after initial diagnosis, but those with ovarian cancer have 22 months median overall survival [12]. For all favorable outcomes, early diagnosis and treatment were integral to the patient's survival [4].

While most patients do not recover from lost neurologic function to any significant degree, they can improve their quality of life and everyday functioning with continued physical/occupational therapy and rehabilitation [13].

## Conclusions

Early recognition and diagnosis of any NPS are extremely important because, in the majority of cases, the paraneoplastic syndrome predates the cancer diagnosis. In the case of paraneoplastic cerebellar degeneration syndromes, such as anti-Yo disease, the devastating neurologic symptoms are typically not reversible, but they may be stopped from progressing further with early treatment of the underlying malignancy. Furthermore, some immune treatments such as plasma exchange, IVIG, and steroids tend to be more effective when instituted early. Additionally, prompt treatment of cancer, when diagnosed in earlier stages, may improve cancer outcomes.

## References

[1] Pelosof LC, Gerber DE (2010). Paraneoplastic syndromes: an approach to diagnosis and treatment. Mayo Clin Proc.

[2] Honnorat J, Antoine JC (2007). Paraneoplastic neurological syndromes. Orphanet J Rare Dis.

[3] De Beukelaar JW, Sillevis Smitt PA (2006). Managing paraneoplastic neurological disorders. Oncologist.

[4] Venkatraman A, Opal P (2016). Paraneoplastic cerebellar degeneration with anti-Yo antibodies - a review. Ann Clin Transl Neurol.

[5] Hu FQ, Shang FR, Liu JJ, Yuan H (2020). Plasma exchange for treating anti-Yo-associated paraneoplastic cerebellar degeneration: case report and literature review. Medicine (Baltimore).

[6] Peterson K, Rosenblum MK, Kotanides H, Posner JB (1992). Paraneoplastic cerebellar degeneration. I. A clinical analysis of 55 anti-Yo antibody-positive patients. Neurology.

[7] Thöne J, Hohaus A, Lamprecht S, Bickel A, Erbguth F (2008). Effective immunosuppressant therapy with cyclophosphamide and corticosteroids in paraneoplastic cerebellar degeneration. J Neurol Sci.

[8] Shams'ili S, de Beukelaar J, Gratama JW, Hooijkaas H, van den Bent M, van 't Veer M, Smitt PS (2006). An uncontrolled trial of rituximab for antibody associated paraneoplastic neurological syndromes. J Neurol.

[9] Widdess-Walsh P, Tavee JO, Schuele S, Stevens GH (2003). Response to intravenous immunoglobulin in anti-Yo associated paraneoplastic cerebellar degeneration: case report and review of the literature. J Neurooncol.

[10] David YB, Warner E, Levitan M, Sutton DM, Malkin MG, Dalmau JO (1996). Autoimmune paraneoplastic cerebellar degeneration in ovarian carcinoma patients treated with plasmapheresis and immunoglobulin. A case report. Cancer.

[11] Rojas-Marcos I, Picard G, Chinchón D (2012). Human epidermal growth factor receptor 2 overexpression in breast cancer of patients with anti-Yo--associated paraneoplastic cerebellar degeneration. Neuro Oncol.

[12] Le May M, Dent S (2018). Anti-Yo antibody-mediated paraneoplastic cerebellar degeneration associated with cognitive affective syndrome in a patient with breast cancer: a case report and literature review. Curr Oncol.

[13] Fu JB, Raj VS, Asher A, Lee J, Guo Y, Konzen BS, Bruera E (2014). Inpatient rehabilitation performance of patients with paraneoplastic cerebellar degeneration. Arch Phys Med Rehabil.

